# Factors associated with a better treatment efficacy among psoriasis patients: a study based on decision tree model and logistic regression in Shanghai, China

**DOI:** 10.1186/s12889-024-19468-9

**Published:** 2024-07-18

**Authors:** Fanlingzi Shen, Zhen Duan, Siyuan Li, Zhongzhi Gao, Rui Zhang, Xiangjin Gao, Bin Li, Ruiping Wang

**Affiliations:** 1 Clinical Research Center, Shanghai Skin Diseases Hospital, School of Medicine, Tongji University, 1278 Baode Road, Jing’an District, Shanghai, 200443 China; 2 School of Public Health, Shanghai University of Traditional Chinese Medicine, 1200 Cai Lun Road, Shanghai, China

**Keywords:** Psoriasis, Treatment efficacy, Tobacco smoking, Decision tree model, Logistic regression

## Abstract

**Background:**

Many effective therapies for psoriasis are being applied in clinical practice in recent years, however, some patients still can’t achieve satisfied effect even with biologics. Therefore, it is crucial to identify factors associated with the treatment efficacy among psoriasis patients. This study aims to explore factors influencing the treatment efficacy of psoriasis patients based on decision tree model and logistic regression.

**Methods:**

We implemented an observational study and recruited 512 psoriasis patients in Shanghai Skin Diseases Hospital from 2021 to 2022. We used face-to-face questionnaire interview and physical examination to collect data. Influencing factors of treatment efficacy were analyzed by using logistic regression, and decision tree model based on the CART algorithm. The receiver operator curve (ROC) was plotted for model evaluation and the statistical significance was set at *P* < 0.05.

**Results:**

The 512 patients were predominately males (72.1%), with a median age of 47.5 years. In this study, 245 patients achieved ≥ 75% improvement in psoriasis area and severity index (PASI) score in week 8 and was identified as treatment success (47.9%). Logistic regression analysis showed that patients with senior high school and above, without psoriasis family history, without tobacco smoking and alcohol drinking had higher percentage of treatment success in patients with psoriasis. The final decision tree model contained four layers with a total of seventeen nodes. Nine classification rules were extracted and five factors associated with treatment efficacy were screened, which indicated tobacco smoking was the most critical variable for treatment efficacy prediction. Model evaluation by ROC showed that the area under curve (AUC) was 0.79 (95%CI: 0.75 ~ 0.83) both for logistic regression model (0.80 sensitivity and 0.69 specificity) and decision tree model (0.77 sensitivity and 0.73 specificity).

**Conclusion:**

Psoriasis patients with higher education, without tobacco smoking, alcohol drinking and psoriasis family history had better treatment efficacy. Decision tree model had similar predicting effect with the logistic regression model, but with higher feasibility due to the nature of simple, intuitive, and easy to understand.

## Background

Psoriasis is a common chronic inflammatory skin disease, often characterized by scaling, erythema and itching [1, 2]. Psoriasis vulgaris is one of the most common subtypes, accounting for 80–90% of psoriasis patients [1, 2]. Epidemiological survey studies have shown that the prevalence of psoriasis in adults ranges from 0.51 to 11.43% [3], and it is estimated that about 125 million people worldwide suffer from psoriasis [1]. China has a huge number of psoriasis patients, about 6.5 million cases [4]. In 2014, psoriasis was recognized as a serious non-communicable disease (NCD) in the World Health Assembly resolution WHA67.9 [5]. Due to the long duration of psoriasis and the fact that it has been shown to affect different organ systems, it brings a great deal of economic pressure and mental burden to the patients, which seriously affects their quality of life [2].

The pathogenesis of psoriasis has not been fully elucidated, and it is currently believed that a combination of genetic and environmental factors is important in triggering or exacerbating the disease [6]. With the development of genome-wide association studies (GWAS), genetic studies have become highly efficient and more than 80 susceptibility loci that play a role in the development of psoriasis have been identified [7, 8]. Environmental influences in psoriasis mainly include ultraviolet radiation, medications, tobacco smoking, alcohol drinking, obesity, infections and stress, which act on the genetic and immune systems to influence the course of the disease [9, 10]. In addition, previous studies have shown that patient’s gender, age, comorbidity and socio-economic characteristics could also affect the severity of psoriasis and the treatment decision-making process [11–13].

In China, psoriasis patients are categorized into mild, moderate and severe according to the severity of the disease. Treatment plans are formulated based on the patient’s condition, comorbidities, personal needs, and affordability. Most patients with mild to moderate psoriasis can be treated with topical medications such as glucocorticoids and vitamin D_3_ derivatives alone; patients with moderate to severe psoriasis can be treated with a combination of systemic medications and physical therapy in addition to topical medications [14]. The emergence of advanced small molecules and biologics has largely improved the efficacy of psoriasis treatment, but there is still no cure for psoriasis. In order to control the condition and reduce symptoms, patients usually need long-term treatment and comprehensive management. Therefore, it is crucial to actively explore modifiable factors influencing the treatment efficacy of psoriasis patients.

Decision tree model is a non-parametric supervised machine learning method, which summarizes the classification rules obtained by systematically learning the attribute features of the existing data [15, 16]. The results are mostly presented in the form of tree diagrams, which is simple, intuitive, and easy to understand. It can be used to quickly identify patients who appear to have an ending event, which is especially suitable in clinical practice [16]. In this study, we aimed to explore factors associated with better treatment efficacy in psoriasis patients based on decision tree model and logistic regression, and apply the CART algorithm to develop a decision tree model to help dermatologists quickly predict treatment efficacy of psoriasis patients.

## Methods

### Study population

We conducted this observational study in Shanghai Skin Diseases Hospital from January 2021 to February 2022. The common influencing factor, tobacco smoking was used as reference for sample size calculation. A previous survey showed the prevalence of tobacco smoking was 25.83% in psoriasis patients in Shanghai [17]. In this study, we applied the sample size calculation formula *n=[µ*_*α*_^*2*^ *× p(1-p)]/δ*^*2*^ and set *p* = 30%, α = 0.05, δ = 15% of p, and a non-response rate of 10%, the sample size calculation indicated that at least 445 psoriasis patients should be recruited. This study was reviewed and approved by the Institutional Review Boards of Shanghai Skin Disease Hospital (2021-44), and was then registered in the Chinese Clinical Trial Registry (registration number: ChiCTR2200066403, date of registration: 05/12/2022). In this study, all psoriasis patients signed the informed consent form before questionnaire interview, and 512 psoriasis patients were finally included in the analysis.

### Diagnosis, inclusion, exclusion criteria of psoriasis

Psoriasis is diagnosed with reference to the Chinese Clinical Dermatology which was in line with the global guidelines for psoriasis diagnosis and treatment [18], i.e., the skin damage is dominated by red inflammatory papules, maculopapular rashes, and plaques of varying sizes covered with multiple layers of silvery-white scales, and scraping off the scales reveals a shiny film with punctate hemorrhages underneath the film. In this study, both of male and female moderate to severe psoriasis patients (body surface area [BSA] ≥ 3, psoriasis area and severity index [PASI] ≥ 3) aged ≥ 18 years in inpatient department were included. The exclusion criteria were as follows: (1) pregnant or lactating women; (2) patients with serious primary diseases such as cardiovascular, cerebrovascular, hepatic, renal, and hematopoietic systems or psychiatric disorders; (3) those with communication disorders who could not complete the questionnaire survey on their own; and (4) those who did not take the medication according to the regulations and could not judge the efficacy, or those who had incomplete data that affected the judgment of efficacy or safety.

### Data collection

In this study, each psoriasis patient would receive the physical examination, BSA, PASI and physician global assessment (PGA) evaluation administrated by dermatologists at their first hospital visit. Then, dermatologists would make a proper treatment plan for each patient and discuss with them, and the individual treatment plan was finally confirmed and implemented through physician-patient shared decision making. In this study, the treatment plan covered four options, acitretin group (25-50 mg daily), methotrexate group (MTX, 15-20 mg per week, with folic acid supplementation), narrow band ultraviolet (NB-UVB)/benvitimod group (2–4 times weekly, plus benvitimod topical treatment concurrently) and biologics group (ustekinumab, risankizumab, secukinumab, etc.). The main contents of the questionnaire were as follows: (1) demographic characteristics: age, gender, education, etc.; (2) medical history of NCD: diabetes mellitus, hypertension, hyperlipidemia, hyperuricemia, coronary atherosclerotic heart disease, non-alcoholic fatty liver disease, tumors, and chronic renal insufficiency; (3) lifestyle habits: tobacco smoking, alcohol drinking, tea drinking, etc.; (4) information on psoriasis severity (BSA, PASI, PGA) at the baseline, week 4 and week 8, seasonality of psoriasis aggravation, family history of psoriasis, and the records of adverse events as well.

### Definition, classification and index calculation

In this study, tobacco smoking was defined as a person who smoked at least 100 cigarettes in lifetime. Alcohol drinking was defined as drinking alcohol at least twice a week for at least six months. Tea drinking was defined as drinking tea at least three times a week for at least six months. Age was stratified into < 50 years and ≥ 50 years. Education was recorded as completed years of schooling and categorized as 0–9 years (junior high school and lower), 10–12 years (senior high school), and > 12 years (college and above). Individual monthly income was categorized as ≤ 5000, 5001–10,000, and > 10,000 (Chinese Yuan, CNY). Marital status was categorized as married and unmarried. Body mass index (BMI) was calculated as weight /height^2^(kg/m^2^) and was categorized as underweight (< 18.5), normal weight (18.5–23.9), overweight (24.0-27.9), and obese (≥ 28.0). Weekly frequency of drinking carbonated beverages (e.g., Coke, Sprite, etc.) was categorized as ≤ 1, 2–3, and ≥ 4. Weekly frequency of consuming high fat foods (e.g., hamburgers, barbecue, etc.) was categorized as ≤ 1, 2–3, and ≥ 4. Daily participation in physical activity was categorized as < 30 min and ≥ 30 min.

PASI quantitatively evaluates the condition of psoriasis in four body regions: head and neck, upper limbs, trunk, and lower limbs, mainly based on the severity of erythema (E), infiltration (I), and desquamation (D) of the patient’s lesions, as well as the area of involvement of the lesions. The formula for PASI score calculation is the head and neck lesion area × (E + I + D)×0.1 + upper limb lesion area × (E + I + D)×0.2 + trunk lesion area × (E + I + D)×0.3 + lower limb lesion area × (E + I + D)×0.4. The PASI formula was used to calculate the total psoriasis score, which ranged from 0 to 72, with higher scores suggesting that patients were more ill [19]. In this study, treatment success was defined as patients who achieved at least 75% improvement in PASI score from baseline, and calculated by the formula [(PASI in baseline -PASI in week t)/PASI in baseline]×100%. The efficacy of treatment was assessed by the percentage of treatment success in week 8 among psoriasis patients.

### Data analysis

In this study, data were analyzed by using SPSS 26.0 (SPSS Inc., Chicago, Illinois, United States). Quantitative variables were expressed as mean and standard deviation (SD) if they conformed to normal distribution, and t-tests were used for comparisons between groups. Quantitative variables were expressed as median and interquartile range (IQR) if they conformed to skewed distribution, and non-parametric rank sum tests were used for comparisons between groups. Qualitative variables were expressed as frequency counts (n) and percentage (%), and chi-square test was used for comparison between groups. In this study, factors that were significant in the univariate analysis were included in the logistic regression model (LRM) and decision tree model (DTM). The LRM analysis was performed with setting the treatment success as the dependent variable and seven previously identified variables as independent variables (Table 1). The LRM was performed using the forward LR method, with *P* < 0.10 as the entry criterion and *P* > 0.15 as the removal criterion. DTM was performed using the CART algorithm. The maximum tree depth was set to four, the minimum number of cases in the parent and child nodes was set to 50 and 10, and split-sample validation (70% for the training set and 30% for the test set) was used. The receiver operator curve (ROC) was plotted for model evaluation, and subgroup analysis was performed to explore the influencing factors of treatment efficacy respectively in each different treatment groups among the psoriasis patients. In this study, the test level was taken as α = 0.05, and the difference was statistically significant when *P* < 0.05 (two-tailed).

**Table 1:** Description of variable assignment

Variable	Assignment description
Treatment efficacy	Patients without success treatment = 1, Patients with success treatment = 2
Age (years)	<50 = 1, ≥ 50 = 2
Gender	Male = 1, Female = 2
Education	Junior high and lower = 1, Senior high = 2, College and above = 3
Psoriasis family history	Yes = 1, No = 2
NCD (non-communicable disease)	Yes = 1, No = 2
Tobacco smoking	Yes = 1, No = 2
Alcohol drinking	Yes = 1, No = 2

## Results

In this study, 512 patients with psoriasis were included in the final analysis. The median age of psoriasis patients was 47.5 years (IQR: 36.0, 61.0). Psoriasis patients were predominately males (369 cases, 72.1%), nearly half (48.1%) of patients had a college and above education, 61.7% had a personal monthly income of more than 5000 CNY, 79.9% were married and more than 60% were overweight or obese. 22.1% of patients had a psoriasis family history and 53.9% of them suffered from NCD. In this study, the percentage of patients with success treatment was 47.9% (245 cases), the treatment success rate was 17.6% in acitretin group, 34.7% in MTX group, 36.6% in NB-UVB/benvitimod group, and 66.1% in biologics group. Moreover, psoriasis patients with age < 50 years, female, higher education, without psoriasis family history, and without NCD comorbidities had higher percentage of treatment success, and the differences were all statistically significant (*P* < 0.05) (Table 2).

**Table 2:** Demographic feature and comorbidities among the 512 psoriasis patients in Shanghai

Variables	All patients (*n* = 512)	Patients with success treatment (*n* = 245)	Patients without success treatment (*n* = 267)	Z/χ^2^	*P*
Age (years), median, IQR	47.5 (36.0, 61.0)	45.0 (34.0, 58.0)	50.0 (37.0, 62.5)	2.56	< 0.01
Age (years), *n* (%)				7.12	< 0.01
< 50	280 (54.7)	149 (53.2)	131 (46.8)		
≥ 50	232 (45.3)	96 (41.4)	136 (58.6)		
Gender, *n* (%)				9.43	< 0.01
Male	369 (72.1)	161 (43.6)	208(56.4)		
Female	143 (27.9)	84 (58.7)	59 (41.3)		
Education, *n* (%)				10.13	< 0.01
Junior high and lower	144 (28.1)	56 (38.9)	88 (61.1)		
Senior high	122 (23.8)	54 (44.3)	68 (55.7)		
College and above	246 (48.1)	135 (54.9)	111 (45.1)		
Monthly income (CNY), *n* (%)				2.59	0.27
≤ 5000	196 (38.3)	85 (43.4)	111 (56.63)		
5001–10,000	201 (39.2)	101 (50.2)	100 (49.8)		
> 10,000	115 (22.5)	59 (51.3)	56 (48.7)		
Marital status, *n* (%)				2.90	0.09
Married	409 (79.9)	188 (46.0)	221 (54.0)		
Unmarred	103 (20.1)	57 (55.3)	46 (44.7)		
BMI (kg/m^2^), *n* (%)				5.01	0.17
< 18.5	22 (4.3)	10 (45.5)	12 (54.5)		
18.5–23.9	182 (35.5)	95 (52.2)	87 (47.8)		
24.0-27.9	200 (39.1)	98 (49.0)	102 (51.0)		
≥ 28.0	108 (21.1)	42 (38.9)	66 (61.1)		
Psoriasis family history, *n* (%)				5.58	0.02
Yes	113 (22.1)	43 (38.1)	70 (61.9)		
No	399 (77.9)	202 (50.6)	197 (49.4)		
NCD, *n* (%)				8.13	< 0.01
Yes	276 (53.9)	116 (42.0)	160 (58.0)		
No	236 (46.1)	129 (54.7)	107 (45.3)		
Diabetes, *n* (%)	77 (15.0)	25 (32.5)	52 (67.5)	8.60	< 0.01
Hypertension, *n* (%)	119 (23.2)	49 (41.2)	70 (58.8)	2.77	0.10
Hyperlipidemia, *n* (%)	100 (19.5)	37 (37.0)	63 (63.0)	5.86	< 0.05
Hyperuricemia, *n* (%)	62 (12.1)	28 (45.2)	34 (54.8)	0.21	0.65
CHD, *n* (%)	27 (5.3)	17 (63.0)	10 (37.0)	2.61	0.11
NAFLD, *n* (%)	112 (21.9)	47 (42.0)	65 (58.0)	1.99	0.16
Tumor, *n* (%)	3 (0.6)	2 (66.7)	1 (33.3)	*-*	0.61
CRI, *n* (%)	3 (0.6)	1 (33.3)	2 (66.7)	*-*	1.00
PASI (baseline), median, IQR	11.2 (8.0, 17.0)	12.1 (8.1, 17.0)	11.0 (7.9, 17.0)	-0.70	0.48
Treatment plan				69.53	< 0.01
Acitretin	68 (13.3)	12 (17.6)	56 (82.4)		
Methotrexate	98 (19.1)	34 (34.7)	64 (65.3)		
NB-UVB/Benvitimod	101 (19.7)	37 (36.6)	64 (63.4)		
Biologics	245 (47.9)	162 (66.1)	83 (33.9)		

*Abbreviations* IQR, interquartile range; CNY, Chinese Yuan; BMI, body mass index; NCD, non-communicable diseases; CHD, coronary atherosclerotic heart disease; NAFLD, non-alcoholic fatty liver disease; CRI, chronic renal insufficiency; PASI, psoriasis area and severity index; NB-UVB, narrow band ultraviolet b

### Differences in treatment efficacy among psoriasis patients with different lifestyle habits

In this study, the prevalence of tobacco smoking and alcohol drinking among 512 psoriasis patients was 43.2% and 24.4%, respectively. Table 3 showed that the percentage of treatment success was higher among psoriasis patients without tobacco smoking and alcohol drinking, and the difference was statistically significant (*P* < 0.05). Nearly 30% of psoriasis patients had tea drinking habit, over 60% of patients drank carbonated beverages ≤ 1 time per week, and 42.4% of patients consumed high fat food 2–3 times per week and 92% of patients had less than 30 min of physical activity per day, but there was no statistically significant difference between them in the aspect of treatment success (Table 3).

**Table 3:** The treatment efficacy among 512 psoriasis patients with different lifestyle habits in Shanghai, China

Variables	All patients (*n* = 512)	Patients with success treatment (*n* = 245)	Patients without success treatment (*n* = 267)	χ^2^	*P*
Tobacco smoking, *n* (%)				110.13	< 0.01
Yes	221 (43.2)	47 (21.3)	174 (78.7)		
No	291 (56.8)	198 (68.0)	93 (32.0)		
Alcohol drinking, *n* (%)				57.48	< 0.01
Yes	125 (24.4)	23 (18.4)	102 (81.6)		
No	387 (75.6)	222 (57.4)	165 (42.6)		
Tea drinking, *n* (%)				0.40	0.53
Yes	151 (29.5)	69 (45.7)	82 (54.3)		
No	361 (70.5)	176 (48.8)	185 (51.2)		
Frequency of drinking carbonated beverages, *n* (%)				5.62	0.06
≤ 1/week	320 (62.5)	151 (47.2)	169 (52.8)		
2–3/week	177 (34.6)	91 (51.4)	86 (48.6)		
≥ 4/week	15 (2.9)	3 (21.4)	12 (78.6)		
Frequency of high fat food consumption, *n* (%)				0.36	0.83
≤ 1/week	162 (31.6)	80 (49.4)	82 (50.6)		
2–3/week	217 (42.4)	104 (47.9)	113 (52.1)		
≥ 4/week	133 (26.0)	61 (45.1)	72 (54.9)		
Physical exercise, *n* (%)				2.27	0.13
< 30 min/day	471 (92.0)	230 (48.8)	241 (51.2)		
≥ 30 min/day	41 (8.0)	15 (36.6)	26 (63.4)		

### Factors associated with treatment efficacy in psoriasis patients based on LRM

**Fig. 1:**
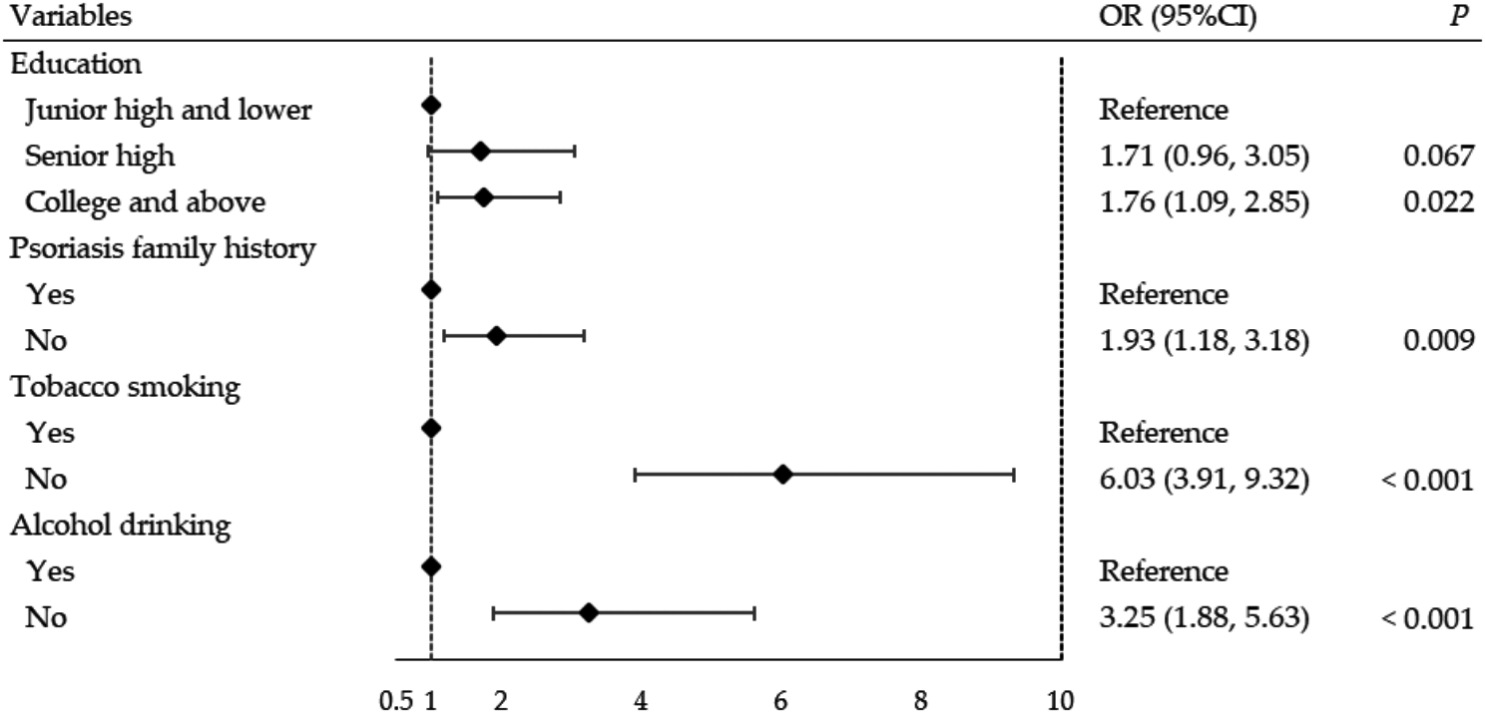
Logistic regression results of treatment efficacy among psoriasis patients

Logistic regression results indicated that the education level, psoriasis family history, tobacco smoking and alcohol drinking were independent factors influencing the treatment efficacy in psoriasis patients. Psoriasis patients with senior high school and college and above education were 1.71 times (OR = 1.71, 95% CI: 0.96–3.05) and 1.76 times (OR = 1.76, 95% CI: 1.09–2.85) more likely to achieve treatment success than those with junior high school and lower education, respectively. Treatment success was more likely to be achieved among those without the psoriasis family history (OR = 1.93, 95% CI: 1.18 ~ 3.18). Moreover, psoriasis patients without tobacco smoking were more likely to have treatment success than those with smoking habit (OR = 6.03, 95% CI: 3.91 ~ 9.32), and psoriasis patients without alcohol drinking also had higher proportion of treatment success (OR = 3.25, 95% CI: 1.88 ~ 5.63) (Fig. 1).

### Factors associated with treatment efficacy in psoriasis patients based on DTM

**Fig. 2:**
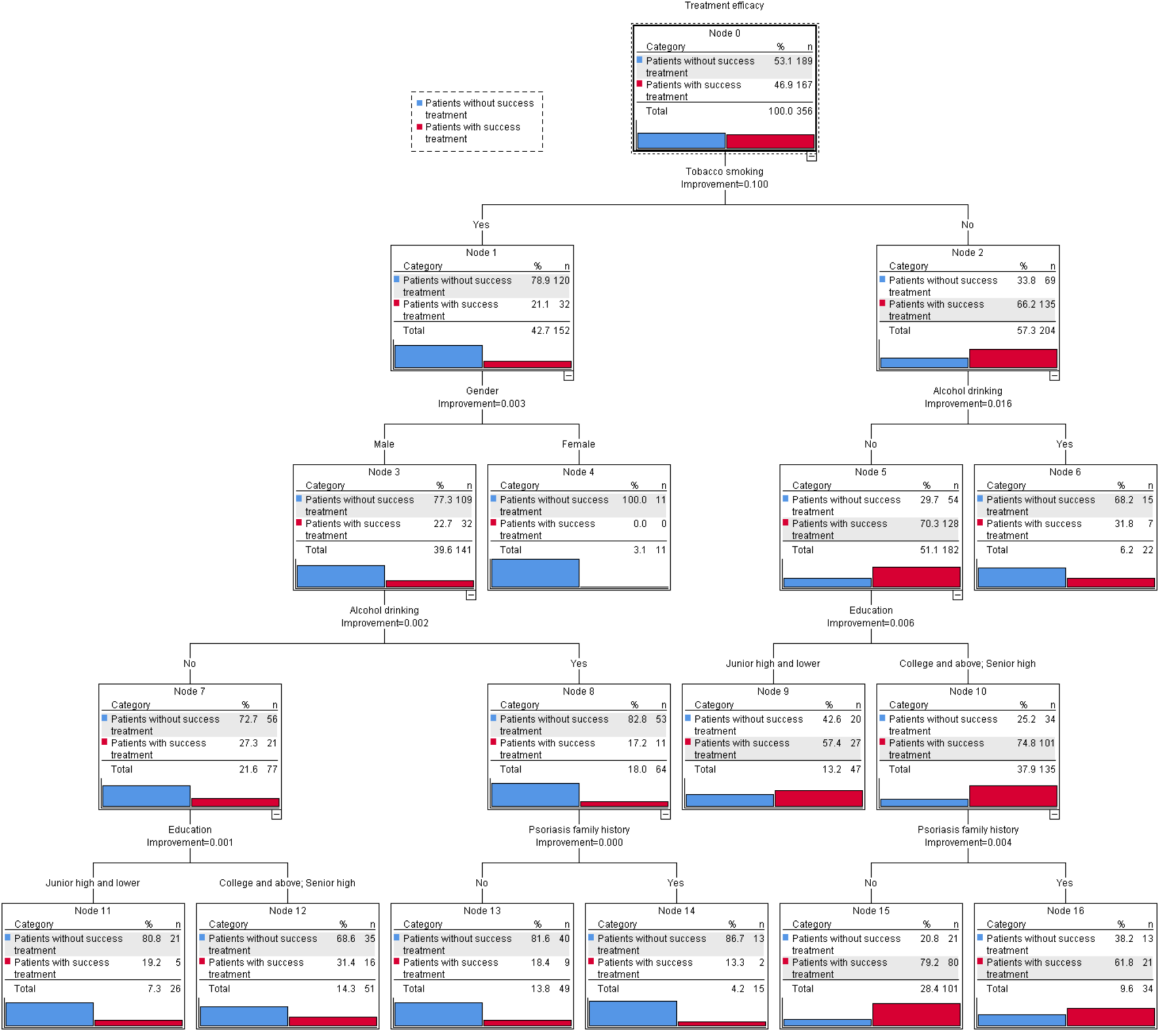
Decision tree model for the treatment efficacy of psoriasis patients

In this study, decision tree model was developed for factors influencing the treatment efficacy among psoriasis patients based on a binary classification tree. The depth of the DTM was four layers, containing one root node, seven internal nodes and nine leaf nodes (Fig. 2). Nine classification rules were extracted (Table 4). Influencing factors that finally entered the DTM were tobacco smoking, alcohol drinking, gender, education, and psoriasis family history. In this study, tobacco smoking was the root node and was identified as the most important factor that negatively associated with the treatment efficacy among psoriasis patients. So psoriasis patients without tobacco smoking had a high probability of achieving successful treatment.

**Table 4:** Decision tree model classification rules for predicting treatment efficacy of the 512 psoriasis patients

Serial number	Node number	Tobacco smoking	Gender	Alcohol drinking	Education	Psoriasis family history	Treatment Success (%)
1	4	Yes	Female	-	-	-	0.0
2	11	Yes	Male	No	Junior high and lower	-	19.2
3	12	Yes	Male	No	Senior high; College and above	-	31.4
4	13	Yes	Male	Yes	-	No	18.4
5	14	Yes	Male	Yes	-	Yes	13.3
6	6	No	-	Yes	-	-	31.8
7	9	No	-	No	Junior high and lower	-	57.4
8	15	No	-	No	Senior high; College and above	No	79.2
9	16	No	-	No	Senior high; College and above	Yes	61.8

### Model evaluation based on ROC for LRM and DTM

**Fig. 3:**
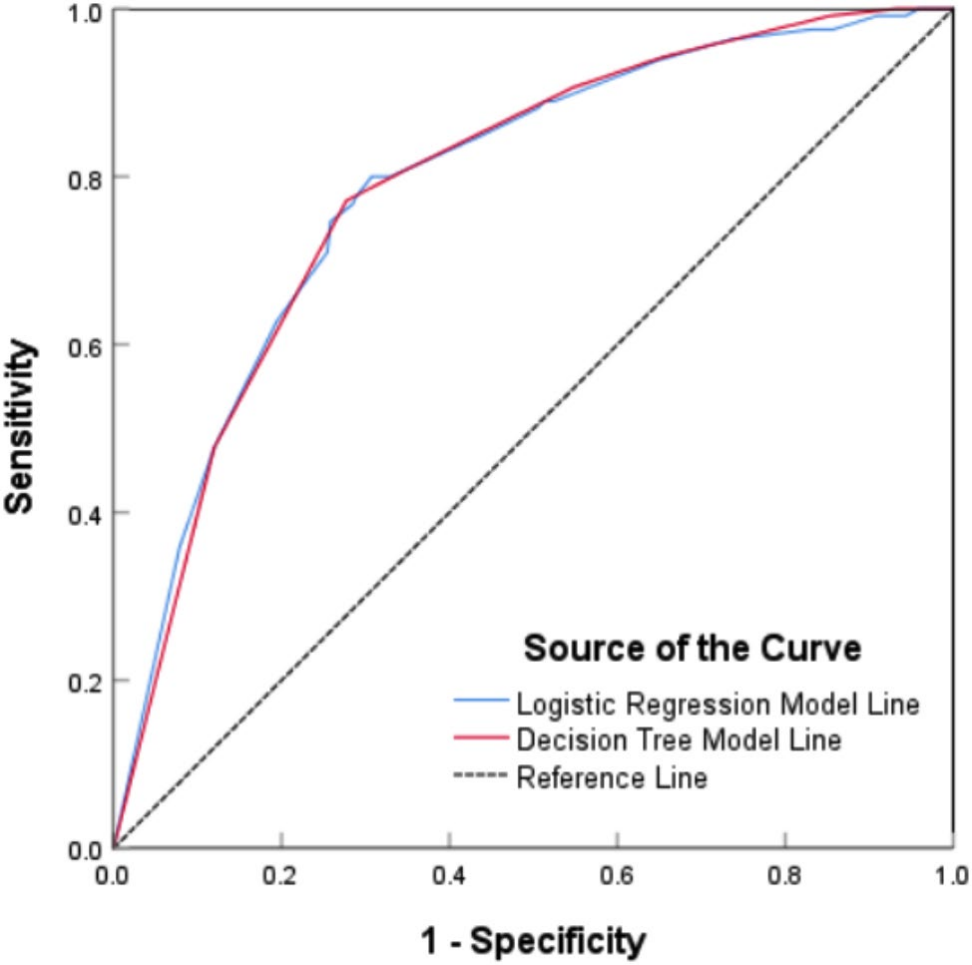
ROC curve results of logistic regression model and decision tree model

In this study, the overall prediction accuracy of the logistic regression model was 74.0%. The prediction accuracy of the decision tree model was 73.9% for the training set and 76.3% for the test set. The prediction probabilities obtained from the logistic regression model and the decision tree model were used as variables to produce the ROC curve plot. Figure 3 showed that the area under curve (AUC) in the ROC for the logistic regression model was 0.79 (95%CI: 0.75 ~ 0.83), with a sensitivity of 0.80 and a specificity of 0.69. The AUC in the ROC curve for the decision tree model was 0.79 (95%CI: 0.75 ~ 0.83), with a sensitivity of 0.77 and a specificity of 0.73. These two ROC curves were similar, indicating that the logistic regression model had similar classification effects as the decision tree model in this study.

### Factors associated with treatment efficacy in different treatment groups

**Fig. 4:**
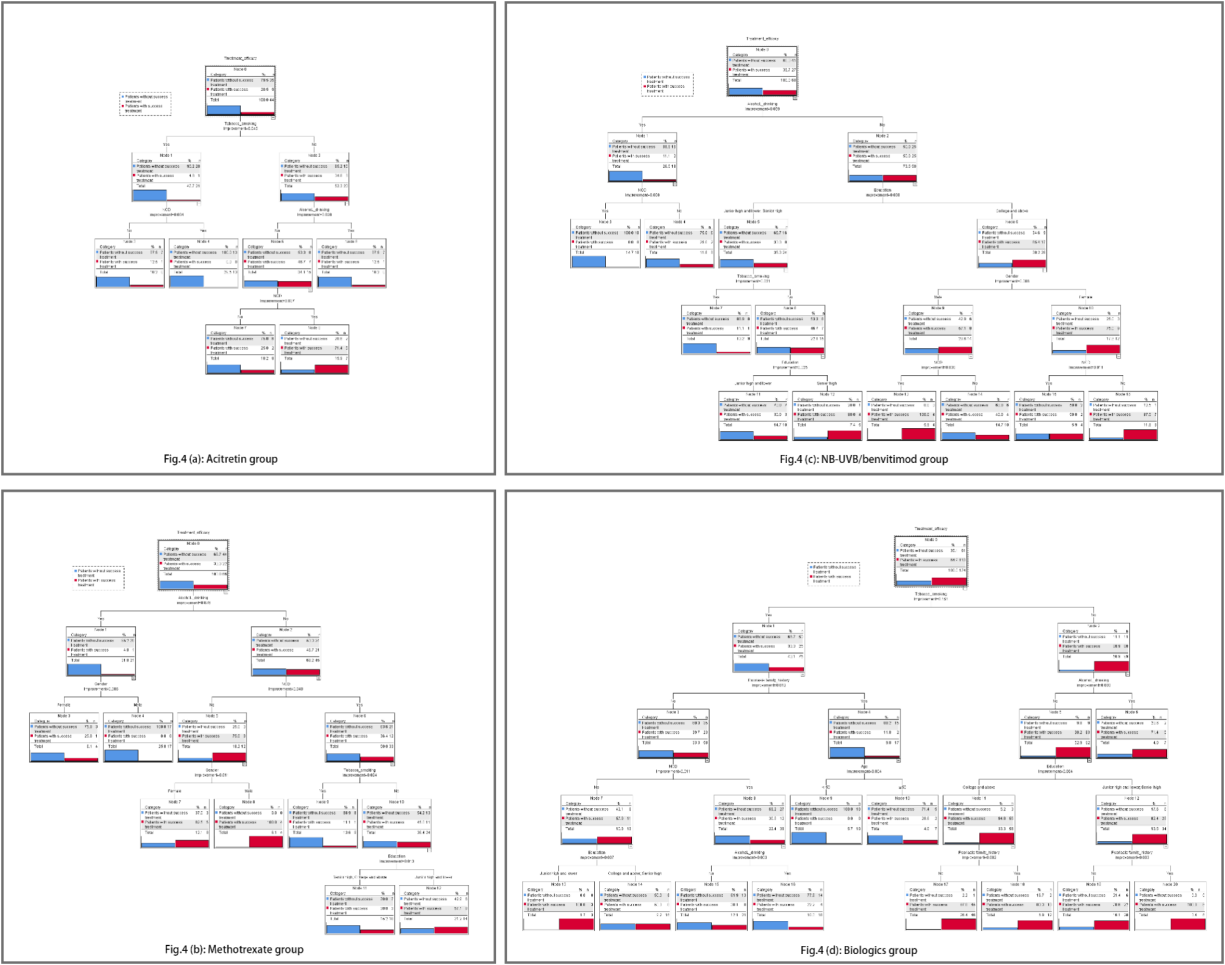
Decision tree model for the treatment efficacy in each treatment groups among psoriasis patients

In this study, 512 psoriasis patients were divided into four treatment groups, the treatment success rate was 17.6% in Acitretin group, 34.7% in Methotrexate (MTX) group, 36.6% in NB-UVB/Benvitimod group, and 66.1% in Biologics group. Logistic regression analysis indicated that tobacco smoking was negatively associated with the treatment success rate (OR = 31.10, 95% CI: 2.40-402.92) in acitretin group. In MTX group, tobacco smoking (OR = 6.11, 95% CI: 1.45–25.73), alcohol drinking (OR = 17.73, 95% CI: 1.77-177.24) negatively affect the treatment success rate. In NB-UVB/benvitimod group, psoriasis patients without tobacco smoking (OR = 4.30, 95% CI: 1.30-14.26), alcohol drinking (OR = 4.76, 95% CI: 1.00-24.31) and with college and above education (OR = 4.77, 95% CI: 1.07–21.25) had higher treatment success rate. In biologics group, tobacco smoking (OR = 13.56, 95% CI: 6.24–29.50) and psoriasis family history (OR = 2.57, 95% CI: 1.16–5.67) negatively affect the treatment success rate. The findings of subgroup analysis in DTM was inline with the LGM (Table 5; Fig. 4).

**Table 5:** Factors associated with the achievement of PASI_75_ in each treatment groups among the 512 psoriasis patients in Shanghai, China

Variables	Model A		Model B		Model C		Model D	
	OR	95% CI	OR	95% CI	OR	95% CI	OR	95% CI
Tobacco smoking								
Yes	1.00	-	1.00	-	1.00	-	1.00	-
No	***31.10***	***2.40-402.92***	***6.11***	***1.45–25.73***	***4.30***	***1.30-14.26***	***13.56***	***6.24–29.50***
Alcohol drinking								
Yes	1.00	-	1.00	-	1.00	-	1.00	-
No	13.24	0.89-196.75	***17.73***	***1.77-177.24***	***4.76***	***1.00-24.31***	1.77	0.79–3.97
Age (years)								
< 50	0.76	0.12–5.01	1.01	0.26–3.97	1.89	0.60-6.00	1.72	0.72–4.12
≥ 50	1.00	-	1.00	-	1.00	-	1.00	-
Gender								
Male	0.39	0.05–3.19	0.97	0.29–3.25	1.13	0.41–3.18	0.66	0.25–1.77
Female	1.00	-	1.00	-	1.00	-	1.00	-
Education								
Junior high and lower	1.00	-	1.00	-	1.00	-	1.00	
Senior high	0.23	0.02–2.56	2.64	0.58–12.13	4.38	0.98–19.54	1.27	0.47–3.46
College and above	0.17	0.02–1.65	0.41	0.11–1.56	***4.77***	***1.07–21.25***	2.03	0.74–5.60
Psoriasis family history								
Yes	1.00	-	1.00	-	1.00	-	1.00	
No	1.88	0.22–16.39	2.17	0.57–3.97	1.34	0.41–4.31	***2.57***	***1.16–5.67***
NCD								
Yes	1.00	-	1.00	-	1.00	-	1.00	-
No	0.50	0.08–3.26	2.19	0.64–7.57	1.54	0.55–4.27	1.59	0.79–3.18

Model A(Acitretin, *n* = 68), Model B (Methotrexate (MTX), *n* = 98), Model C (NB-UVB/Benvitimod, *n* = 101), Model D (Biologics, *n* = 245)

OR: odds ratio; CI: confidence interval; NCD: non-communicable diseases; NB-UVB: narrow band ultraviolet b

The significance of bold values in this table are as follows: Model A: Tobacco smoking: *P* = 0.009; Model B: Tobacco smoking: *P* = 0.014; Alcohol drinking: *P* = 0.014; Model C: Tobacco smoking: *P* = 0.017; Alcohol drinking: *P* = 0.050; Education (college and above): *P* = 0.040; Model D: Tobacco smoking: *P* = 0.001; Psoriasis family history: *P* < 0.014

## Discussion

To our knowledge, this is the first study which recruited over 500 psoriasis patients to explore factors associated with the treatment efficacy based on the real clinical practice data in China. The findings in this study suggest that tobacco smoking, alcohol drinking, psoriasis family history, and lower level of education are negatively associated with the treatment efficacy among psoriasis patients. In the decision tree model, tobacco smoking is the root node and the most important factor to predict the treatment success among psoriasis patients.

In this study, the prevalence of tobacco smoking among psoriasis patients was 43.2%, which was higher than that in previous studies [20]. Meanwhile, data in this study showed that the proportion of treatment success in psoriasis patients without tobacco smoking (68.0%) was significantly higher than that in patients with smoking (21.3%), which was in line with previous studies. A meta-analysis by Zhou et al. [21] concluded that psoriasis patients with previous tobacco smoking were less likely to have an improvement in their PASI condition at 6 months after the treatment with biologics compared to those without tobacco smoking (OR = 0.80, 95% CI: 0.67 to 0.95). Tobacco produces a large number of free radicals during combustion, which activate several signaling pathways that adversely affect the condition of psoriasis; in addition, nicotine stimulates the body to overproduce pro-inflammatory cytokines, which are involved in the formation of psoriatic lesions [22]. The impact of smoking on psoriasis is complex, not only affecting the occurrence of psoriasis, but also closely related to the severity of the disease, co-morbidities and treatment outcomes [23–25]. Therefore, physicians should ask patients about their smoking status and provide plausible advice on smoking cessation in the process of clinical treatment to improve their diseases condition and the treatment efficacy.

Previous studies indicate that alcohol drinking attenuates the efficacy of psoriasis treatment [26–28]. A prospective, multi-center cohort study in the United Kingdom showed that alcohol binge drinking was significantly associated with poor response to psoriasis treatment (*β* = 1.40, *P* < 0.05) [26]. In this study, alcohol drinking was an important internal node in the decision tree model which determined the treatment efficacy of psoriasis patients. Logistic regression analysis also showed that patients without alcohol drinking were 3.25 times more likely to achieve treatment success than those with alcohol drinking, suggesting that alcohol drinking was a barrier for the treatment success. Alcohol drinking may exacerbate the inflammatory process among psoriasis patients through increasing the production of tumor necrosis factor (TNF)-α and enhancing the activity of pro-inflammatory cytokines, which lead to the deterioration of psoriasis [29]. Alcohol drinking may also affect patients’ treatment choices, adherence, and treatment efficacy [29]. So, alcohol drinking is detrimental to patients with psoriasis, and clinicians should enhance health education among psoriasis patients and advise them to abstain from alcohol drinking during the treatment process to promote symptomatic relief and improve the treatment efficacy.

Previous studies have shown that patients with a psoriasis family history tend to have an earlier onset and longer duration of the disease and affect the phenotype and severity of the disease [30, 31], suggesting that genetic factors negatively influence the progression of the disease. Moreover, patients with a psoriasis family history tend to have poorer treatment adherence than those without a family history, which may be related to the presence of negative attitudes towards long-term treatment and disease prognosis among their family members with psoriasis [32]. In this study, 22.1% patients reported a psoriasis family history, which was similar to the findings of previous studies [33]. Patients without a psoriasis family history had a higher probability of maintaining a clinical response after the end of treatment [34]. This study demonstrated that patients without a psoriasis family history were more likely to achieve treatment success than those with a psoriasis family history. So we suggest dermatologists should consider the diversity of conditions and treatment adherence among psoriasis patients with a psoriasis family history and make a plausible treatment plan to improve their compliance in treatment and improve their treatment efficacy.

In this study, approximately 50% patients had college and above education, which was similar with previous studies [12]. Previous studies have shown that less educated patients have a higher chance of having more severe skin lesions [35]. In this study, logistic regression and decision tree analysis suggested that patients’ education level was positively associated with the treatment efficacy. Psoriasis patients in senior high school, college and above were more likely to achieve treatment success than those in junior high school and below, which might due to the fact that psoriasis patients with higher education tend to pay more attention to their health condition and make better compliance with their treatment. This might also relate to the fact that patients with higher education have more financial resources that assist them to choose treatments with better efficacy.

Previous studies have indicated that male psoriasis patients have older age of diseases initiation, with longer diseases course, and more NCD comorbidities [12], while female patients have more disturbances in terms of mental health and quality of life [36]. In this study, female psoriasis patients had a higher proportion of treatment success than male patients, and the difference was statistically significant. But with the adjustment of other variables in logistic regression model, there was no significant difference in efficacy between male and female, which was in line with previous studies [37, 38]. Decision tree analysis showed that the proportion of successful treatment of female patients with psoriasis in smokers was lower than that in male, suggesting that female patients were more susceptible to the effects of smoking, but the mechanisms behind this need to be further investigated [39].

In this study, we did not find a relationship between individual monthly income, BMI, tea drinking, frequency of drinking carbonated beverages, frequency of high fat food consumption, physical exercise and treatment efficacy. Scala et al. [11] showed that patients with lower income levels were less likely to receive biological agents. Previous studies have also shown that patients with psoriasis eat more dairy products and soft drinks than those in the control group without psoriasis, and this study found that eating high-calorie and high-fat foods such as instant noodles increased the severity of psoriasis [40]. There are also studies have shown that moderate or high-intensity physical activity can reduce the severity of psoriasis and improve the quality of life, but despite this, patients with psoriasis still have less physical activity [41]. Therefore, we still recommend the combination of dietary intervention, exercise and psoriasis drug therapy to reduce inflammation and enhance immunity.

A strength of this study was the longitudinal design for data collection among 512 enrolled patients during their treatment in the clinics, and the 8 weeks of follow-up ensured a 100% compliance among psoriasis patients. Moreover, clinical data of patients with psoriasis were extracted from the Health Information System (HIS) directly without recall bias, which ensued the high data quality, is another strength of this study.

This study has some limitations. First of all, all patients are enrolled in one hospital, which ensures the high internal authenticity, but the generalization of the findings is limited. Secondly, our questionnaire survey collects information on the lifestyle habits of psoriasis patients through face-to-face interviews, which may have recall bias. Thirdly, the time point for treatment efficacy assessment is set as in week 8, but week 12 is more commonly used for treatment efficacy assessment, so the treatment success in week 8 in this study has limited comparability with other studies, and the incorporation of more assessment time points should be considered. Fourthly, the duration of treatment observed in all patients was the same (8 weeks), which limited us to explore the relationship between the duration of treatment and the treatment efficacy of psoriasis. Fifthly, patients in this study received different types of drug treatment, which may have impact on the results of the study.

## Conclusions

Psoriasis patients with higher education, without tobacco smoking, alcohol drinking and psoriasis family history had better treatment efficacy. Decision tree model had similar predicting effect with the logistic regression model, but with higher feasibility due to the nature of simple, intuitive, and easy to understand. We recommend dermatologists can use the decision tree model to predict the treatment efficacy of psoriasis patients, and provide timely guidance and health education concerning lifestyle change to improve the treatment efficacy.

## Data Availability

The data for this study are available upon request from the corresponding author. The request should state the title and aim of the research for which the data are requested.

## Abbreviations

AUC: Area Under Curve
BMI: Body Mass Index
BSA: Body Surface Area
CART: Classification and Regression Tree
CI: Confidence Interval
CNY: Chinese Yuan
DTM: Decision Tree Model
GWAS: Genome-Wide Association Studies
HIS: Health Information System
IQR: Interquartile Range
LRM: Logistic Regression Model
MTX: Methotrexate
NB-UVB: Narrow Band Ultraviolet B
NCD: Non-communicable Disease
OR: Odds Ratio
PASI: Psoriasis Area and Severity Index
PGA: Physician Global Assessment
ROC: Receiver Operator Curve
SD: Standard Deviation
TNF: Tumor Necrosis Factor
